# Isolation of highly enriched primary human microglia for functional studies

**DOI:** 10.1038/srep19371

**Published:** 2016-01-18

**Authors:** Justin Rustenhoven, Thomas I-H Park, Patrick Schweder, John Scotter, Jason Correia, Amy M. Smith, Hannah M. Gibbons, Robyn L. Oldfield, Peter S. Bergin, Edward W. Mee, Richard L. M. Faull, Maurice A. Curtis, E. Scott Graham, Mike Dragunow

**Affiliations:** 1 Department of Pharmacology and Clinical Pharmacology, 1023, Auckland, New Zealand; 2 Centre for Brain Research, 1023, Auckland, New Zealand; 3 Department of Anatomy with Radiology, 1023, Auckland, New Zealand; 4 Lab Plus, Auckland City Hospital, Auckland, New Zealand; 5 Auckland City Hospital, 1023, Auckland, New Zealand

## Abstract

Microglia, the resident macrophages of the central nervous system play vital roles in brain homeostasis through clearance of pathogenic material. Microglia are also implicated in neurological disorders through uncontrolled activation and inflammatory responses. To date, the vast majority of microglial studies have been performed using rodent models. Human microglia differ from rodent counterparts in several aspects including their response to pharmacological substances and their inflammatory secretions. Such differences highlight the need for studies on primary adult human brain microglia and methods to isolate them are therefore required. Our procedure generates microglial cultures of >95% purity from both biopsy and autopsy human brain tissue using a very simple media-based culture procedure that takes advantage of the adherent properties of these cells. Microglia obtained in this manner can be utilised for research within a week. Isolated microglia demonstrate phagocytic ability and respond to inflammatory stimuli and their purity makes them suitable for numerous other forms of *in vitro* studies, including secretome and transcriptome analysis. Furthermore, this protocol allows for the simultaneous isolation of neural precursor cells during the microglial isolation procedure. As human brain tissue is such a precious and valuable resource the simultaneous isolation of multiple cell types is highly beneficial.

The brain was once believed to be an immune privileged organ, implying it could tolerate antigen introduction without eliciting an inflammatory response^1^. The identification of brain resident macrophages, termed microglia, challenged this long held view^2^,^3^. In the mammalian brain microglia represent approximately 12% of the total cell number, however, this can vary significantly between regions (5–20%)^2^,^4^. As the brain’s classical immune cell, microglia extend long, mobile processes which constantly survey the central nervous system (CNS) microenvironment. This enables microglia to detect any perturbations in the brain and subsequently respond to many forms of insult^5^. On recognition of an insult, microglia become “activated” and demonstrate enhanced phagocytic ability, as well as the production of inflammatory cytokines/chemokines and neurotrophic factors. Ultimately this process attempts to restore CNS homeostasis through the removal of invading pathogens, dead cells and debris, hence promoting neuronal survival^6^. Whilst an acute microglial-mediated immune response appears necessary for correct CNS functioning, uncontrolled chronic microglial activation is believed to precipitate neuronal damage and subsequent death. As such, microglia are often referred to as a double-edged sword^6^.

A growing body of evidence suggests that there are significant differences between rodent and human microglia, both in their biochemistry and responsiveness to pharmacological substances. For example, rodent microglia can be stimulated to secrete large quantities of nitric oxide, a reactive oxidative species responsible for a significant amount of microglial-mediated neurotoxicity, however, this function appears either absent or significantly blunted in human microglia^7^. Furthermore, rodent microglia display opposing effects with respect to alterations in phagocytic function and apoptotic cell death when treated with the anti-epileptic drug valproic acid^8^,^9^. These differences highlight the need for primary human microglia with respect to understanding their basic functioning, as well as in drug discovery.

This paper describes a method to isolate primary human microglia from both biopsy and autopsy brain tissue. Microglial cultures generated in this manner display a high degree of purity that makes them suitable for almost all forms of *in vitro* studies, including examination of inflammatory cytokine/chemokine secretion and phagocytosis, two critical functions of these cells *in vivo*, as well as whole population transcriptome and secretome analysis.

## Results

### Characterisation of culture purity

**Figure 1:**
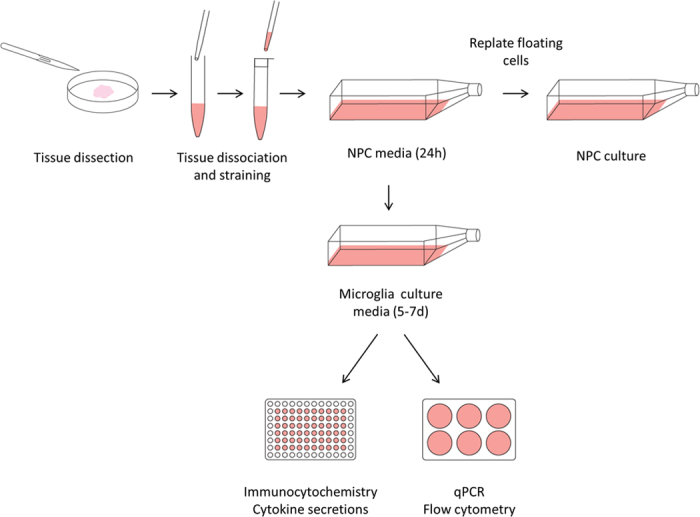
Overview of isolation procedure. Human brain biopsy or autopsy tissue was mechanically and enzymatically dissociated to achieve a single cell suspension. This suspension was strained and plated in neural precursor cell media for up to 24 hours. Floating and weakly attached cells were removed and replated into a new flask and used to generate neural precursor cell cultures. Microglial culture media was added to the original flask for 5–7 days and microglia were harvested and utilised for experimental procedures.

**Figure 2:**
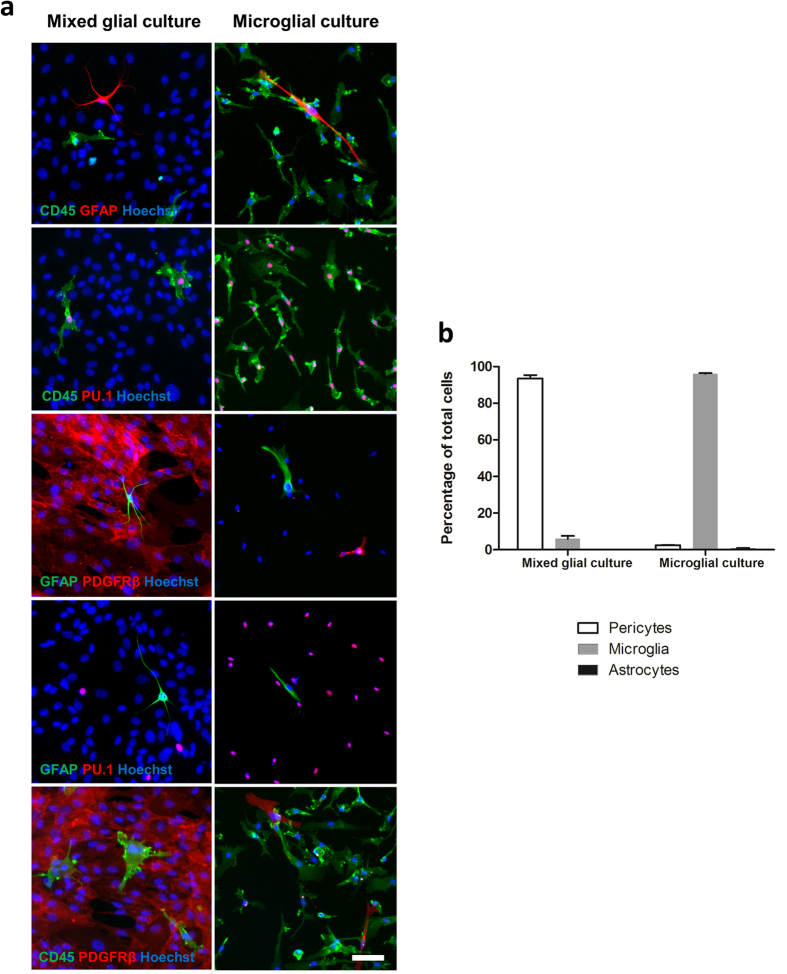
Characterisation of culture purity. Culture purity of our standard mixed glial isolation and microglial specific isolation was determined via immunocytochemistry using markers specific for microglia (CD45, PU.1), pericytes (PDGFRβ) and astrocytes (GFAP) (**a**). The percentages of each cell type were determined (**b**). N = 3 for mixed glial culture isolation (all epilepsy), N = 7 (three epilepsy, three GBM and one Parkinson’s disease) for microglia isolation. Scale bar = 50 μm.

The protocol described below (summarized in Fig. 1) produced high purity microglial cultures from adult human brain tissue derived from epilepsy and glioblastoma multiforme (GBM) surgeries as well as one Parkinson’s disease autopsy case of short post-mortem delay. Regarding the cultures from GBM surgeries the cells generated were likely a mix of microglia and macrophages but appeared similar to epilepsy derived microglia (Fig. S1). Immunocytochemistry data (Fig. 2a,b) shows representative images and quantification of epilepsy-derived microglia, however, a similar purity with elevated yields was observed from GBM samples whilst autopsy tissue produced microglia cultures of similar purity, but with decreased yields (data not shown).

As demonstrated by immunocytochemical analysis, mixed glial cultures show relatively low abundance of microglia (5.97 ± 1.59% CD45/PU.1 positive) compared to pericytes (93.92 ± 1.76% PDGFRβ positive). A small amount of astrocytes are also present (0.11 ± 0.03% GFAP positive; Fig. 2a,b). When cultured using the microglial isolation procedure the microglial cultures are highly enriched (96.75 ± 0.39% CD45/PU.1 positive; Fig. 2a,b). Small amounts of contaminating cells remain present including pericytes (2.43 ± 0.18% PDGFRβ positive) and astrocytes (0.82 ± 0.11% GFAP positive; Fig. 2a,b). Microglia cultured from the same tissue sample using either the mixed glial culture protocol or the microglia culture protocol displayed similar purity and yields as above, confirming that the aforementioned results are due to the processing of the cultures and not tissue heterogeneity (Fig. S2). Markers were chosen based on our previous experience with cell types obtained from human biopsy tissue. The relative contribution of microglia, astrocytes and pericytes equates to 100% suggesting that there are no other contaminating cell types.

### Isolated microglia express typical microglial markers

**Figure 3:**
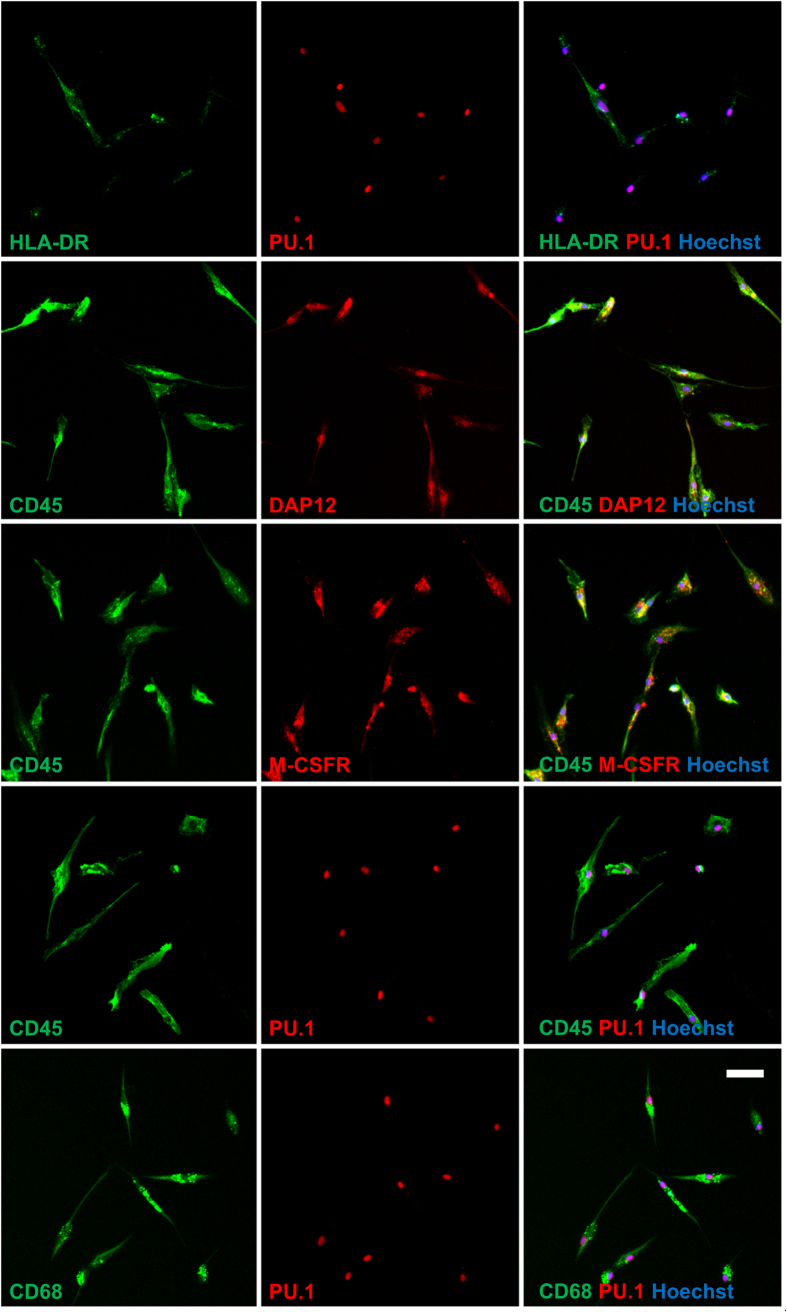
Isolated microglia express typical microglial markers. Isolated microglia were found to express markers present on microglia derived from mixed glial cultures. All CD45 or PU.1 positive cells co-expressed CD68, M-CSFR, DAP12 whilst displaying variable levels of HLA-DR. Representative images from three (all epilepsy) independent cases are shown. Scale bar = 50 μm.

In order to confirm that the phenotype of the isolated PU.1/CD45 positive cells reflected that of human microglia grown in a mixed glial culture, a more detailed immunocytochemical analysis was performed. Like primary human microglia grown as mixed cultures^10^, isolated microglia displayed heterogeneous levels of HLA-DP, DQ, DR, but overall this expression was low indicating an un-activated state (Fig. 3). Furthermore, each PU.1 or CD45 positive cell was found to co-express CD68, M-CSFR or DAP12, typical microglial markers both *in vitro* and *in vivo* (Fig. 3). In order to compare the phenotype of isolated microglia to those in a mixed glial culture, these experiments were only performed on epilepsy-derived microglia. Currently we do not know enough about human GBM-derived microglia/macrophages to determine whether potential differences in phenotype are a disease-related or protocol-related phenomenon.

### Isolated microglia express cytoplasmic NF-κB under basal conditions which can be translocated to the nucleus by IL-1β and LPS but not IFNγ

**Figure 4:**
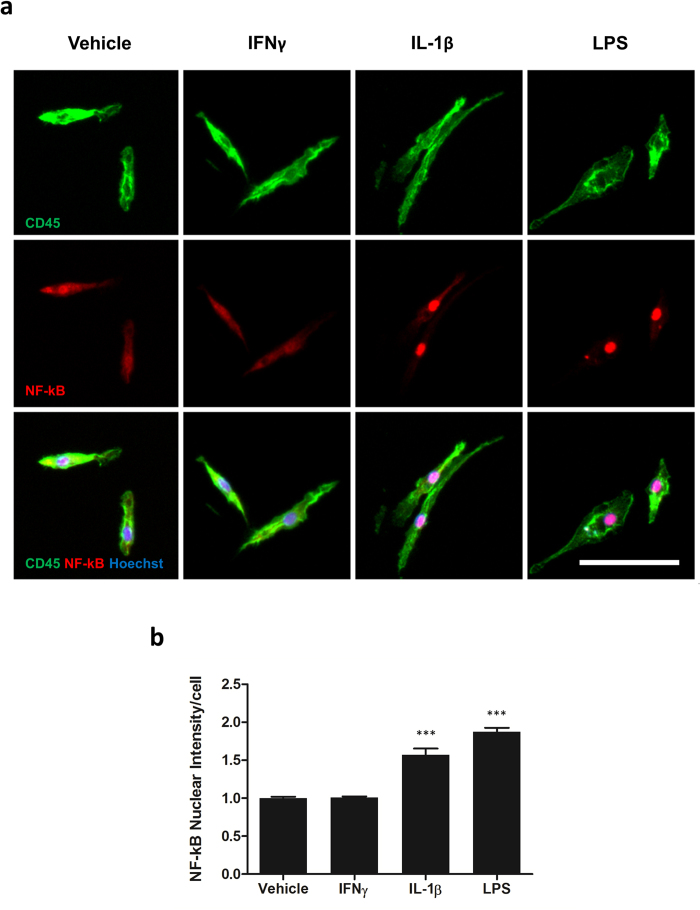
Isolated microglia express cytoplasmic NF-κB under basal conditions which can be translocated to the nucleus by IL-1β and LPS but not IFNγ. Microglia were treated with 10 ng/mL IL-1β, LPS, IFNγ or vehicle (0.1% BSA in PBS) for one hour and immunostained for NF-kB and CD45 (**a**). The intensity of nuclear NF-kB was determined (**b**). N = 3 (all epilepsy). Scale bar = 50 μm. ***p < 0.001.

In order to evaluate the activation state of microglia, immunocytochemical staining of NF-kB p65 was performed. As previously described in our mixed glial cultures, microglia isolated from epilepsy tissue display both cytoplasmic and nuclear NF-kB under basal conditions^11^. Both IL-1β and LPS significantly enhance nuclear translocation of NF-kB whilst IFNγ does not (Vehicle = 1.00 ± 0.02 AU, IFNγ = 1.01 ± 0.02 AU; p > 0.05, IL-1β = 1.57 ± 0.08 AU; p < 0.001, LPS = 1.88 ± 0.05 AU; p < 0.001; Fig. 4) indicating that microglia can be further activated by inflammatory stimuli.

### Microglial HLA-DR, DP, DQ expression is induced by IFNγ but not IL-1β or LPS

**Figure 5:**
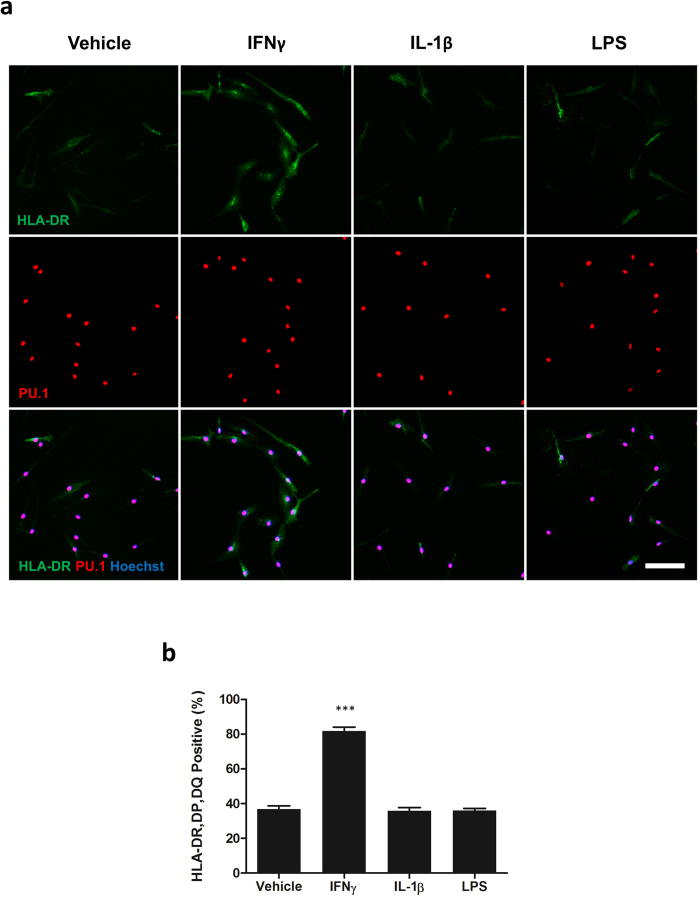
Microglial HLA-DR, DP, DQ expression is induced by IFNγ but not IL-1β or LPS. Microglia were treated with 10 ng/mL IL-1β, LPS, IFNγ or vehicle (0.1% BSA in PBS) for 24 hours and immunostained for HLA-DR, DP, DQ and PU.1 (**a**). The percentage of HLA, DR, DP, DQ positive cells was determined (**b**). N = 3 (two epilepsy and one GBM). Scale bar = 50 μm. ***p < 0.001.

To further examine the activation state of microglia and confirm a functional response to immune stimuli, HLA-DR, DP, DQ expression was examined in unstimulated cells or cells treated with IL-1β, IFNγ or LPS. Under basal conditions HLA-DR, DP, DQ expression was present in 36.80 ± 1.92% of microglia and was not significantly affected by IL-1β (35.80 ± 1.87%; p > 0.05) or LPS (36.03 ± 1.30%; p > 0.05). However, IFNγ significantly enhanced HLA-DR, DP, DQ expression (81.67 ± 2.374%; p < 0.001), highlighting their stimulus-dependent inflammatory response (Fig. 5).

### Isolated microglia can phagocytose fluorescent beads

**Figure 6:**
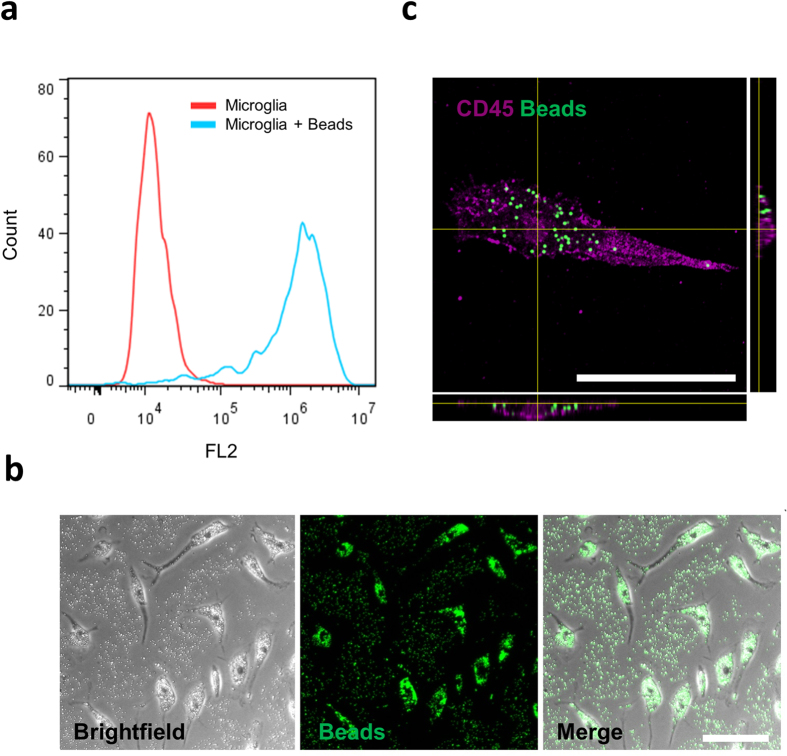
Isolated microglia can phagocytose fluorescent beads. Microglia were cultured in the presence or absence of fluorescent beads (1 μm diameter) for two hours. Cells were washed thoroughly to remove un-internalized beads and collected by trypsinisation and gentle scraping. Phagocytosis of beads was determined by a rightward shift in FL2 intensity via flow cytometry (**a**). One representative plot from three (two epilepsy and one GBM, frontal cortex) independent experiments is shown. Brightfield and fluorescent imaging of cells immediately prior to trypsinisation shows the distribution of beads within cells (**b**). Confocal scanning laser microscopy confirmed that microglia were able to internalise beads (**c**). Scale bar = 50 μm.

A critical function of microglia *in vivo* is the removal of apoptotic cells, cellular debris and pathogenic material by phagocytosis. In order to determine whether isolated microglia maintain their phagocytic function, the ability to phagocytose latex beads, a commonly used assay with respect to microglia, was utilised. Flow cytometry based assays provide a quick and powerful measurement of phagocytic ability. When incubated with fluorescent latex beads for 2 hours, microglia produced a significant rightward shift in fluorescence (FL2) intensity, indicative of internalized beads. More than 90% of microglia where found to have phagocytosed beads during this time indicating that microglia in this manner are suitable for studies investigating phagocytosis (Fig. 6a,b). In order to confirm that beads were internalized by cells and not simply bound to the cell surface, confocal microscopy was performed (Fig. 6c)

### Measurement of cytokine/chemokine secretion from isolated microglia

**Figure 7:**
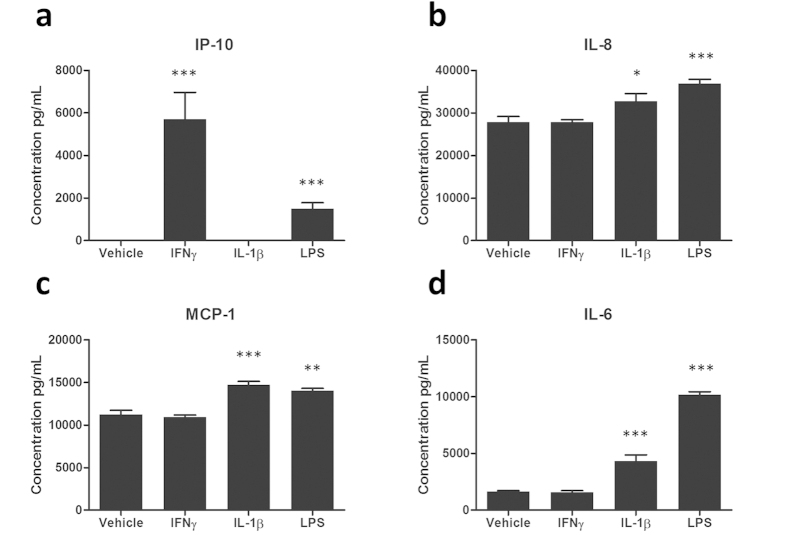
Measurement of cytokine/chemokine secretion from isolated microglia. Microglia were treated with 10 ng/mL IL-1β, IFNγ, LPS or vehicle (0.1% BSA in PBS) for 24 hours. Conditioned media was collected and concentrations of IP-10 (**a**), IL-8 (**b**), MCP-1 (**c**) and IL-6 (**d**) determined in triplicate wells by a multiplex cytometric bead array. One representative case from three (all epilepsy) independent experiments is shown. *p < 0.05, **p < 0.01, ***p < 0.001.

Microglia secrete a range of inflammatory mediators aimed to regulate the brains immune response. Isolated microglia from epilepsy tissue express IL-6 (1,614 ± 109 pg/mL), MCP-1 (11,223 ± 524 pg/mL) and IL-8 (25,455 ± 1,378 pg/mL) basally whilst IP-10 was absent (0 ± 0 pg/mL; Fig. 7a–d). IP-10 secretions were present following IFNγ (5,706 ± 1,258 pg/mL; p < 0.001) and LPS stimulation (1,502 ± 288 pg/mL; p < 0.001) whilst IL-1β treatment showed no induction (0 ± 0 pg/mL; p > 0.05; Fig. 7a). A significant induction of IL-8 was seen with IL-1β (32,727 ± 1,831 pg/mL; p < 0.05) and LPS (35,396 ± 1,012 pg/mL; p < 0.001), however, IFNγ displayed no change (27,854 ± 552 pg/mL; p > 0.05; Fig. 7b). MCP-1 was significantly elevated by IL-1β (14,703 ± 414 pg/mL; p < 0.001) and LPS (14,025 ± 281 pg/mL; p < 0.01) with IFNγ having no effect (10,935 ± 241 pg/mL; p > 0.05; Fig. 7c). IL-6 secretions were significantly enhanced by IL-1β (4,324 ± 550 pg/mL; p < 0.001) and LPS (10,174 ± 253 pg/mL; p < 0.001) whilst IFNγ showed no induction (1,560 ± 163 pg/mL; p > 0.05; Fig. 7d). Notably, the secretion profile of microglia varied significantly with differing stimuli. Microglia isolated in this manner are therefore suitable for studies investigating inflammatory mediator secretions.

## Discussion

Mixed glial cultures containing pericytes, microglia and astrocytes have been routinely used in our laboratory to examine microglial biology^10^,^11^,^12^,^13^,^14^. While these mixed cultures have the benefit of more accurately capturing the CNS environment, particularly the neurovascular unit, the presence of multiple cell types complicates data obtained for experiments relating specifically to microglial function. Primary human microglia isolated using the protocol described here allow for investigations into changes in cell secretions using cytometric bead arrays, flow cytometry-based analysis of phagocytosis and microglial gene expression by qRT-PCR. Each of which is complicated in mixed cultures due to the ability of pericytes and astrocytes to contribute to the observed responses.

An important step in the development of this protocol was to determine whether microglia cultured in this manner appear, and indeed behave, the same as microglia in mixed glial cultures. Isolated cells expressed typical ramified microglia morphology, characteristic of ‘resting’ microglia, as well as displaying the human microglial markers CD45, PU.1, CD68, M-CSFR, DAP12 and HLA-DR^10^,^13^,^14^. The relatively low level of HLA-DR expression and the cytoplasmic NF-κB expression suggests that the isolation procedure did not fully activate the microglia. However, microglia remained immunologically active and demonstrated nuclear translocation of NF-kB with IL-1β and LPS, as well as induction of HLA-DR, DP, DQ with IFNγ alone, as described previously for microglia grown as mixed cultures^10^,^11^. Furthermore, microglia produced an enhanced secretion of the inflammatory mediators IL-6, MCP-1, IP-10 and IL-8 with immunogenic stimuli. Interestingly the secretome profile was found to differ between stimuli. Whilst microglia retained the ability for phagocytosis, a typical function both *in vivo* and *in vitro,* this was not altered by inflammatory state (Fig. S4). Whilst pro-inflammatory mediators have been suggested to increase microglia phagocytosis, we have previously shown that macrophage-colony stimulating factor (M-CSF), a mitogen which polarizes microglia to an anti-inflammatory state, enhanced phagocytosis of microglia in a mixed glial culture^13^.

Numerous other protocols exist for the isolation of (typically rodent) microglia. The majority of studies use either a Percoll® gradient followed by an extended centrifugation to isolate the microglial fraction^15^,^16^,^17^, extensive shaking of the flask to prevent attachment of other cells^18^, or sorting via magnetic or fluorescence activated cell sorting (MACS/FACS)^17^,^19^. However, previous attempts in our lab to isolate human microglia using MACS sorting produced initially pure microglia but at very low yields (Fig. S3). Our attempts to isolate human microglia via FACS resulted in impure cultures of activated microglia with contaminating pericytes quickly taking over cultures (unpublished observations). In contrast, using the protocol described in this paper we are able to isolate relatively large numbers of pure adult human microglia, with the added advantage of also isolating separately, human neural precursor cells (NPC’s). It is our belief that isolation and study of human microglia using a variety of methods is important so that comparisons of microglial function can be made using different isolation protocols to determine true microglial functions.

As described above, another major advantage of our protocol is the ability to generate NPC cultures in parallel. This methodology was originally established for the generation of NPC’s from neurogenic regions of the adult human brain as well as GBM specimens and isolation of microglia was a serendipitous discovery. NPC isolation and subsequent differentiation can be performed using the cells removed from the flask after day one as described previously^20^. In order to generate NPC’s from human brain tissue, growth factors (EGF and FGF2) and heparin are added to culture media. Importantly, these are solely for the benefit of NPC culture. Microglia cultured in the presence or absence of these factors for the first 24 hours showed no differences with respect to yield, phenotype or inflammatory state (Fig. S5). Furthermore, when cultured as a mixed glial culture, microglia do not require these growth factors for survival^21^. However, as human brain biopsy tissue is such a precious and valuable resource we chose to use these growth factors in each culture, in order to maximise the possible cell types obtained.

It should be noted that microglia cultured in this manner do not have a high proliferation rate *in vitro.* While other groups have used M-CSF to enhance microglial proliferation in culture, this mitogen significantly alters microglial biology, particularly receptor expression and phagocytic function, and so was avoided in our protocol^10^,^13^.

Whilst this protocol routinely and reliably produces healthy human microglia, several steps are important with respect to minimizing variation. On the second day (no longer than 24 hours) of culturing when unattached cells are removed, the plate should be thoroughly washed to prevent any cells other than microglia remaining. Other potential cells that can remain are astrocytes and pericytes. Whilst astrocytes do not proliferate readily *in vitro,* pericytes do and if not sufficiently removed will quickly take over cell cultures. To further avoid pericyte contamination media changes should be kept minimal; often no change in media is needed. It is also important to allow microglia to settle sufficiently in the flask such that they begin to extend processes. The time for this to occur does vary, but if cells are detached too soon they do not reattach well, resulting in reduced yields. Lastly, once plated out, microglia quickly adhere and therefore should be used as soon as possible, preferably experiments should be started the next day. To account for the possibility of contaminating cells, a small number of wells should be plated out each time for characterisation of culture purity.

Using the current protocol, we routinely obtain >95% pure microglia cultures from human brain that can be used to perform highly accurate experiments to interrogate microglial functions *in vitro*, including cellular phenotyping, transcriptome analysis, cytokine/chemokine release and phagocytosis. It should be noted that although we observed a similar microglia purity whether the tissue was derived from biopsy epilepsy or biopsy GBM surgeries, we did not investigate likely phenotypic differences between microglia derived from these different sources. This will form the basis of our future studies. However, the markers that we did use to identify microglia (CD45, PU.1) were expressed at similar levels for all cultures (Fig. S1).

## Methods

### Tissue Source

Biopsy human brain tissue was obtained, with informed written consent, from the middle temporal gyrus, anterior temporal lobe and hippocampus of three patients undergoing surgery for medically refractive epilepsy, or frontal cortex from two patients and temporal cortex from one patient with glioblastoma multiforme (GBM) resection. Autopsy tissue with a short post-mortem delay (2.5 hours) was obtained from the middle temporal gyrus and subventricular zone of a single patient with Parkinson’s disease. Autopsy tissue with a longer post-mortem delay (17.5 hours) was obtained from the middle temporal gyrus and subventricular zone of a single patient with Parkinson’s disease, however, yielded no viable microglia. All protocols used in this study were approved by the Northern Regional Ethics Committee (New Zealand) for biopsy tissue, and the University of Auckland Human Participants Ethics committee for the post-mortem brain tissue. All methods were carried out in accordance with the approved guidelines.

### Microglial isolation from human brain tissue biopsies

Following surgical resection, tissue was transported to the research facility. Approximately 1–2 g of tissue was washed in HBSS, and meninges and visible blood vessels were removed. Tissue was diced into pieces approximately 1 mm^3^ using a sterile scalpel and transferred to a 50 mL falcon tube containing 10 mL enzyme dissociation mix (10 U/mL DNase (Invitrogen, CA, USA) and 2.5 U/mL papain (Worthington, NJ, USA) in Hibernate-A medium (Gibco, CA, USA)) per gram of tissue for 10 minutes at 37 °C with gentle rotation. The tissue was removed from the incubator, gently triturated to aid digestion and returned to the incubator for a further 10 minutes. Dissociation was slowed by adding an equal volume of Dulbecco’s modified eagle medium: Nutrient mixture F-12 (DMEM/F12; Gibco, CA, USA) with 1% B27 (Gibco, CA, USA) and the cell suspension was passed through a 70 μm cell strainer (Bector Dickinson, NJ, USA). Cells were centrifuged at 160 × g for 10 minutes, the supernatant discarded and resuspended in 20 mL neural precursor cell (NPC) proliferation media (DMEM/F12 with 1% B27, 1% GlutaMAX (Gibco, CA, USA), 1% penicillin-streptomycin-glutamine (PSG; Gibco, CA, USA), 40 ng/mL fibroblast growth factor-2 (FGF-2; Peprotech, NJ, USA), 40 ng/mL epidermal growth factor (EGF; Peprotech, NJ, USA) and 2 μg/mL heparin (Sigma, MO, USA)). The cell suspension was transferred to a T75 tissue culture flask (Nunc, Roskilde, Denmark) and incubated overnight at 37 °C with 95% air/5% CO_2_. The following day the tissue culture flask was tapped firmly to remove non-adherent or loosely-adherent cells and these were transferred to a new T75 tissue culture flask. This flask contains predominantly NPC’s, pericytes and astrocytes which can be used as described previously^12^,^20^. The original flask containing the adherent cells was washed twice with NPC proliferation media and 15 mL of microglial culture media was added (DMEM/F12 with 10% foetal bovine serum (FBS; Moregate, QLD, Australia) and 1% PSG). Microglia were maintained in this media for up to 1 week at 37 °C with 95% air/5% CO_2_ with minimal media changes to prevent contaminating cell growth. Once microglia begin to extend processes they can be utilised for further studies. When cultured as described above, microglial yields of 2–300,000 cells/gram of tissue can be expected from epilepsy biopsy samples. GBM biopsies were significantly more varied, but substantially higher than that of epilepsy surgeries. Yields for autopsy samples are also extremely variable and highly dependent on the post-mortem delay. One Parkinson’s disease autopsy sample with a short post mortem delay (2.5 hours) resulted in a yield of ~100,000 cells/gram whilst another Parkinson’s case with a long post mortem delay (17.5 hour hours) produced no viable microglia. An overview of the microglial isolation procedure is summarized in Fig. 1.

### Mixed glial isolation from human brain tissue

Mixed glial cultures containing astrocytes, pericytes and microglia were isolated from biopsy adult human brain tissue and cultured for 1–2 weeks before plating as described previously^12^,^21^.

### Cell plating

To harvest cells for plating, culture media was removed and T75 tissue culture flasks were washed with phosphate buffered saline (PBS). 3 mL of 0.25% trypsin-1 mM ethylenediaminetetraacetic acid (EDTA; Gibco, CA, USA) was added for five minutes at 37 °C with 95% air/5% CO_2_. Microglia attach firmly to the T75 tissue culture flasks and to aid microglial detachment cells were gently scraped with a rubber cell scraper (Falcon, MA, USA). Trypsin was neutralised by addition of microglia culture media and cells counted using a haemocytometer. Cells were plated at 5,000 cells/well for 96 well plates or 25,000 cells/well for 24 well plates. For confocal experiments cells were plated on glass coverslips inside a 48 well plate at 5,000 cells/well. Cells were allowed to attach overnight before utilisation for experiments.

### Immunogen treatment

To investigate microglial inflammatory responses cells were treated for 1–24 hours with 10 ng/mL LPS (from *Escherichia coli* 026:B6, L4391, Sigma, MO, USA), IFNγ (R&D Systems, MN, USA), IL-1β (Peprotech, NJ, USA) or vehicle (0.1% BSA in PBS).

### Immunocytochemistry

Cells were fixed in 4% paraformaldehyde (Scharlau, Spain) for 15 minutes and washed in PBS with 0.1% triton X-100 (PBS-T; Sigma, MO, USA). Cells were incubated overnight with antibodies (Table S1) diluted in immunobuffer (PBS with 0.2% triton X-100, 1% goat serum (Gibco, CA, USA) and 0.04% thimerosal (Sigma, MO, USA)). Cells were washed twice in PBS-T and incubated overnight at 4 °C with gentle agitation with appropriate anti-species secondary antibodies diluted in immunobuffer (Table S1). Nuclei were counterstained with Hoechst 33258 for 30 minutes and washed in PBS-T. Images were acquired using an automated fluorescent microscope (ImageXpress® Micro XLS; Version 5.3.0.1, Molecular Devices, CA, USA). Quantitative analysis of intensity measures and positively stained cells was performed using the cell scoring and integrated morphometry analysis modules on MetaXpress® software (Version 5.3.0.1, Molecular Devices, CA, USA). Roughly 1000–2000 cells were scored per well with multiple wells (at least three) analysed per sample.

### Flow cytometry phagocytosis assay

To investigate the ability of microglia to internalize particles they were incubated for two hours in the presence or absence of a 1:1,000 dilution of 1 μm diameter fluorescent beads (Fluoresbrite® YG Carboxylate microspheres; Polysciences Inc, PA, USA). At completion cells were washed twice with PBS to remove un-internalized beads and collected into a single cell suspension by trypsinisation and gentle scraping as per microglial plating. Samples were run on an Accuri C6 flow-cytometer (BD Biosciences, CA, USA) and viable cells gated based on forward scatter and side scatter. Fluorescent intensity of live cells was indicative of the amount of beads phagocytosed.

### Confocal laser scanning microscopy

To confirm internalization of beads for phagocytosis assays, confocal imaging was performed. Microglia were plated on glass coverslips as described above and incubated with a 1:10,000 dilution of 1 μm diameter fluorescent beads (Fluoresbrite® YG Carboxylate microspheres; Polysciences Inc, PA, USA) for 24 hours. Coverslips were immunostained as described under immunocytochemistry and mounted onto glass slides using fluorescent mounting medium (DAKO, Denmark). Confocal images were acquired at a 63 x magnification (1.4 NA) in a Z-series with a gap of 0.8 μm using a Zeiss LSM 710 inverted confocal microscope (Biomedical Imaging Research Unit, University of Auckland).

### Cytometric bead array

Conditioned media (70 μL) was collected from cells grown in a 96 well plate. To remove possible floating cells and debris, media was spun at 160 ×g for five minutes and 40 μL was transferred to a new tube and stored at −20 °C. The concentration of secreted cytokine/chemokines (Table S2) was determined using a multiplexed cytometric bead array (CBA; BD Biosciences, CA, USA) run on an Accuri C6 flow cytometer (BD Biosciences, CA, USA) as described previously^22^,^23^. Data was analysed using FCAP-array software (Version 3.1; BD Biosciences, CA, USA) to convert fluorescent intensity values into concentrations.

### Statistical analysis

Unless otherwise stated, all experiments were performed at least three independent times from three different tissue donors. Statistical analysis was carried out using one-way analysis of variance (ANOVA) followed by Dunnett’s multiple comparison test to compare treatments versus vehicle control (Graphpad Prism 5.02). For growth factor experiments a Two-way ANOVA with Bonferroni post–test was used to compare between treatments.

## Additional Information

**How to cite this article**: Rustenhoven, J. *et al*. Isolation of highly enriched primary human microglia for functional studies. *Sci. Rep.* **6**, 19371; doi: 10.1038/srep19371 (2016).

## Supplementary Material

Supplementary Information
