# Supplementary material for: Isolation of highly enriched primary human microglia for functional studies
Source: Sci Rep. 2016 Jan 18;6:19371. doi: 10.1038/srep19371 (PMC4725991; doi:10.1038/srep19371)
Supplement: Supplementary Information [file srep19371-s1.doc]

**Isolation of highly enriched primary human microglia for functional studies**

Justin Rustenhoven^1,2*^, Thomas I-H Park^1,2,3*^, Patrick Schweder^2,5^, John Scotter^5^, Jason Correia^5^, Amy M. Smith^2^, Hannah M. Gibbons^2^, Robyn L. Oldfield^4^, Peter S. Bergin^2,5^, Edward W. Mee^2,5^, Richard L. M. Faull^2,3^, Maurice A. Curtis^2,3^ , E. Scott Graham^1,2^ and Mike Dragunow^1,2^.

Table S1. List of antibodies used for ICC

| Antigen | Source | Catalogue # | ICC dilution |
| --- | --- | --- | --- |
| Mouse anti-CD68 | Abcam | AB955-500 | 1:500 |
| Rabbit anti-M-CSFR | Santa Cruz | SC-692 | 1:100 |
| Mouse anti-HLA,DR,DP,DQ | DAKO | M0775 | 1:500 |
| Rabbit anti-DAP12 | Santa Cruz | SC-20783 | 1:500 |
| Mouse anti-CD45 | Abcam | Ab8216 | 1:500 |
| Rabbit anti-PU.1 | Cell Signalling | 2258 | 1:500 |
| Rabbit anti-PDGFRβ | Cell Signalling | mAb3169 | 1:500 |
| Rabbit anti-NF-kB P65 | Santa Cruz | SC-372 | 1:500 |
| Rabbit anti-GFAP | DAKO | Z0334 | 1:50,000 |
| Goat anti-mouse Alexa Fluor® 488 | Invitrogen | A11001 | 1:500 |
| Goat anti-mouse Alexa Fluor® 594 | Invitrogen | A11005 | 1:500 |
| Goat anti-rabbit Alexa Fluor® 488 | Invitrogen | A11008 | 1:500 |
| Goat anti-rabbit Alexa Fluor® 594 | Invitrogen | A11012 | 1:500 |

**Table S2. CBA flex sets**

| Antibody | Catalogue # | Bead position |
| --- | --- | --- |
| IL-6 | 558276 | A7 |
| IL-8 | 558277 | A9 |
| IP-10 | 558280 | B5 |
| MCP-1 | 558287 | D8 |


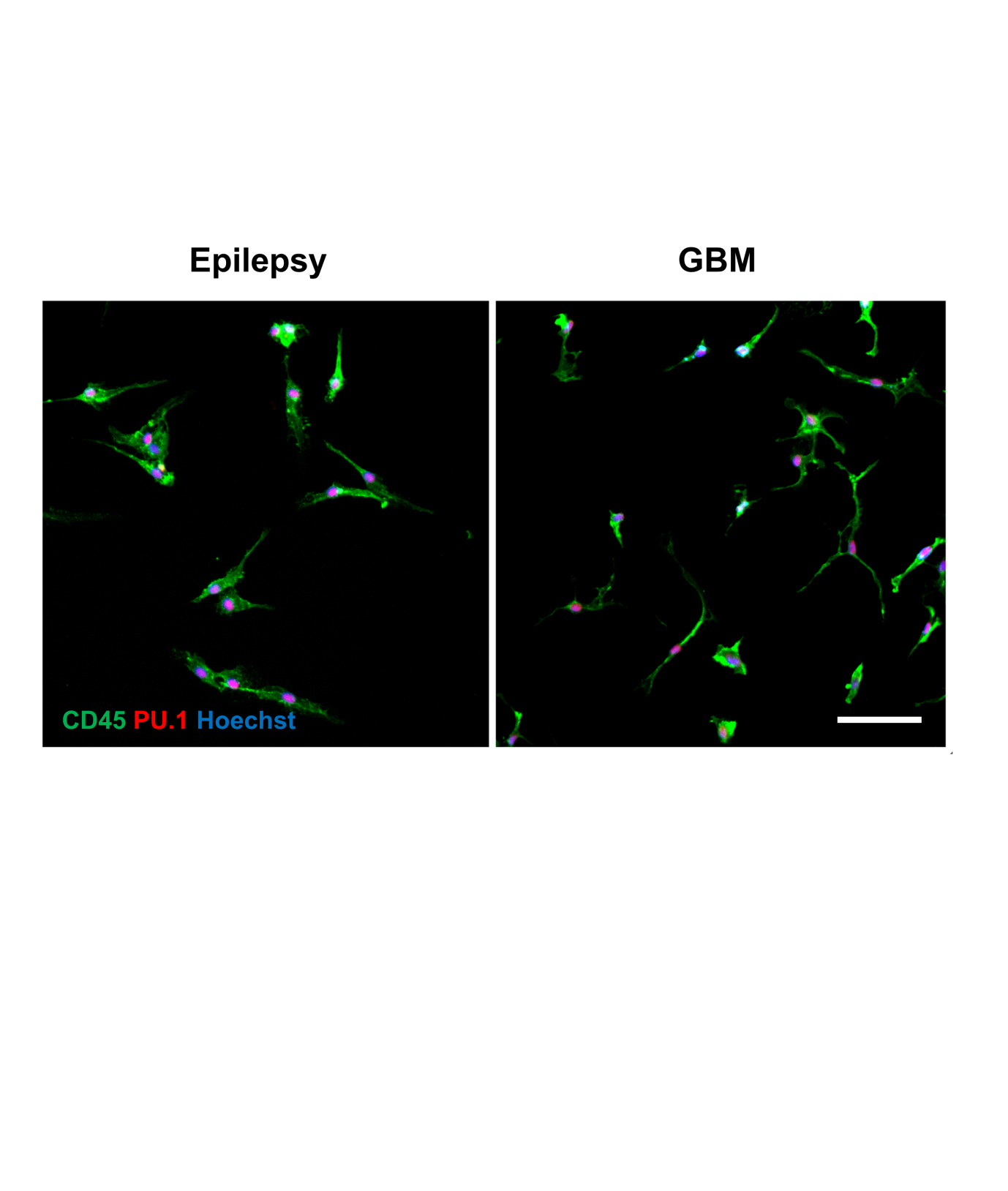


**Figure S1. GBM and epilepsy derived microglia both display CD45 and PU.1 expression**. Microglia were isolated from the MTG of epilepsy biopsy tissue or the frontal lobe of GBM biopsy tissue. Cells were immunostained for microglial markers CD45 and PU.1. Nuclei were counterstained with Hoechst. Scale bar = 50µm.


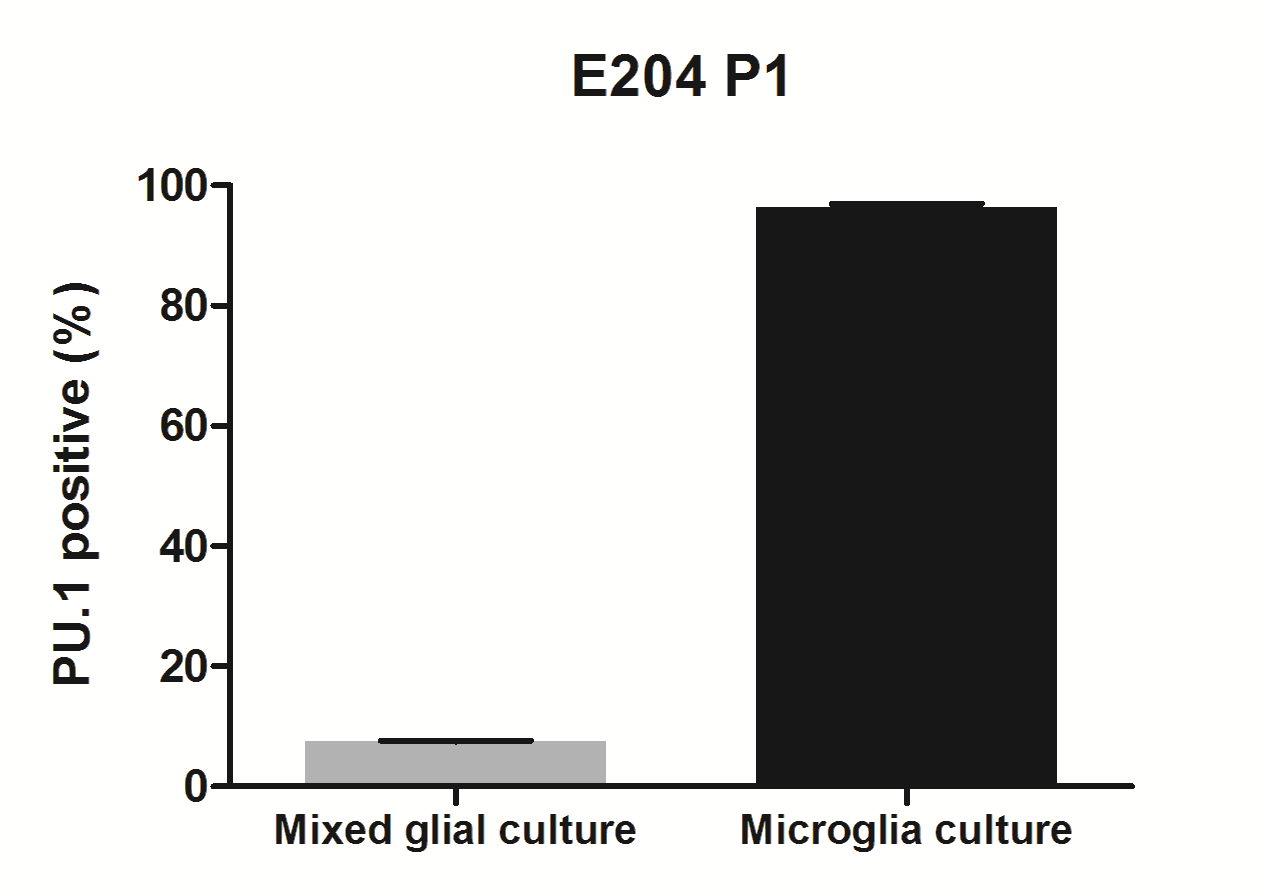


**Figure S2. Characterisation of microglial purity from a single biopsy sample.** Middle temporal gyrus epilepsy tissue was cultured according to either the mixed glial culture protocol or the microglial culture protocol. The percentage of microglia, as determined by PU.1 positivity, was determined at passage two using each method.


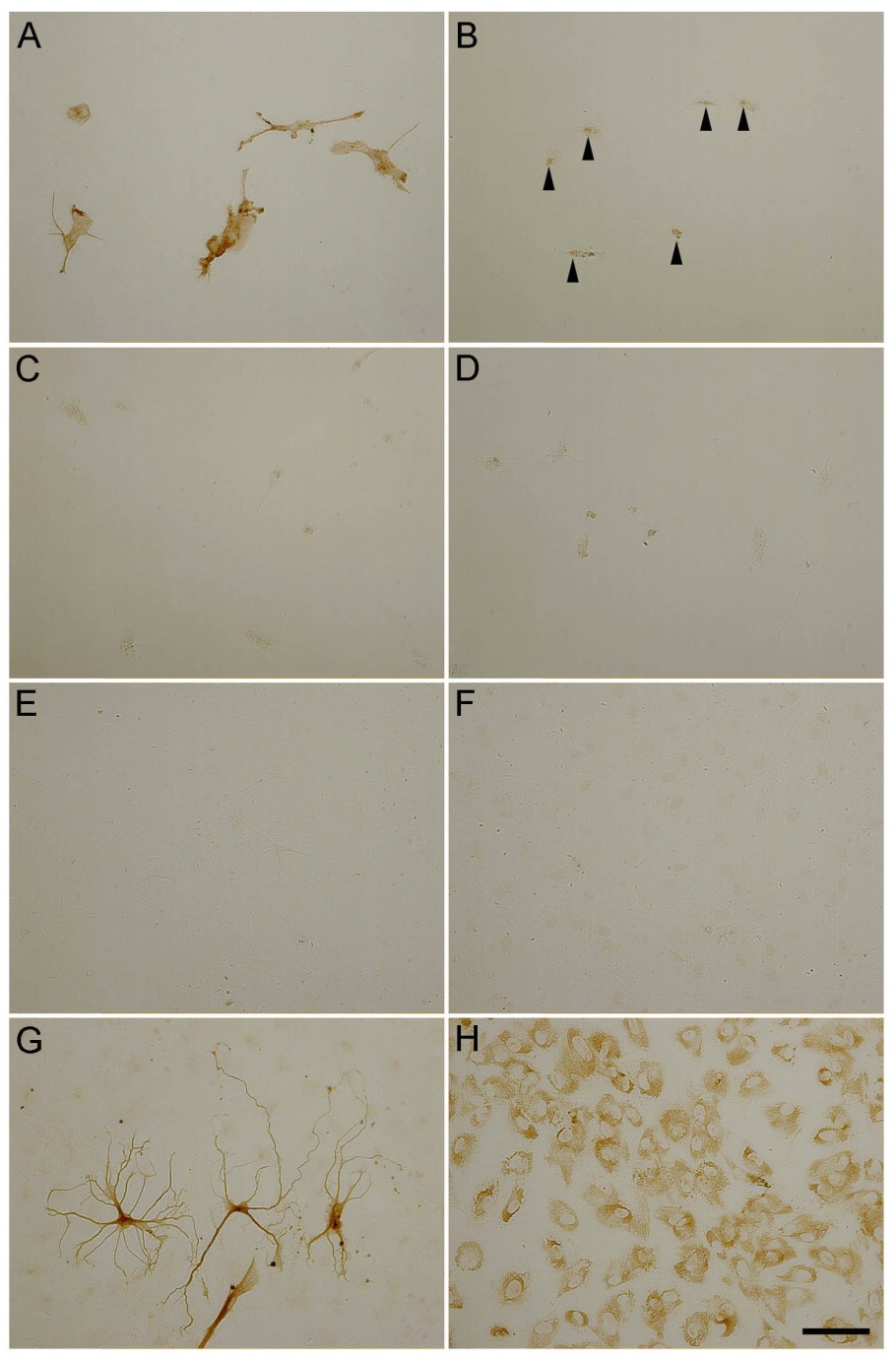


**Figure S3. MACS-CD45 positive selection for human microglia.** Middle temporal gyrus epilepsy tissue was initially treated using the mixed glial isolation protocol (Ref 11). A suspension of mixed glia was prepared and incubated with mouse anti-human CD45 IgG1 isotype for 5 minutes at 4^o^C, washed by centrifugation and then incubated with MACS rat anti-mouse IgG1 microbeads for 15 minutes at 4^o^C. Cells were again washed by centrifugation and then passed through a MACS separation unit column. The column was washed and the positive cell fraction containing CD45-positive microglia eluted (A-D). The negative fraction was also collected for analysis (E-H). Cells were then plated for characterisation and immunostained for CD45 (A, E), PU.1 (B, F, arrow heads in B), GFAP (C, G), and prolyl-4-hydroxylase (D, H). Scale bar = 100µm.

**
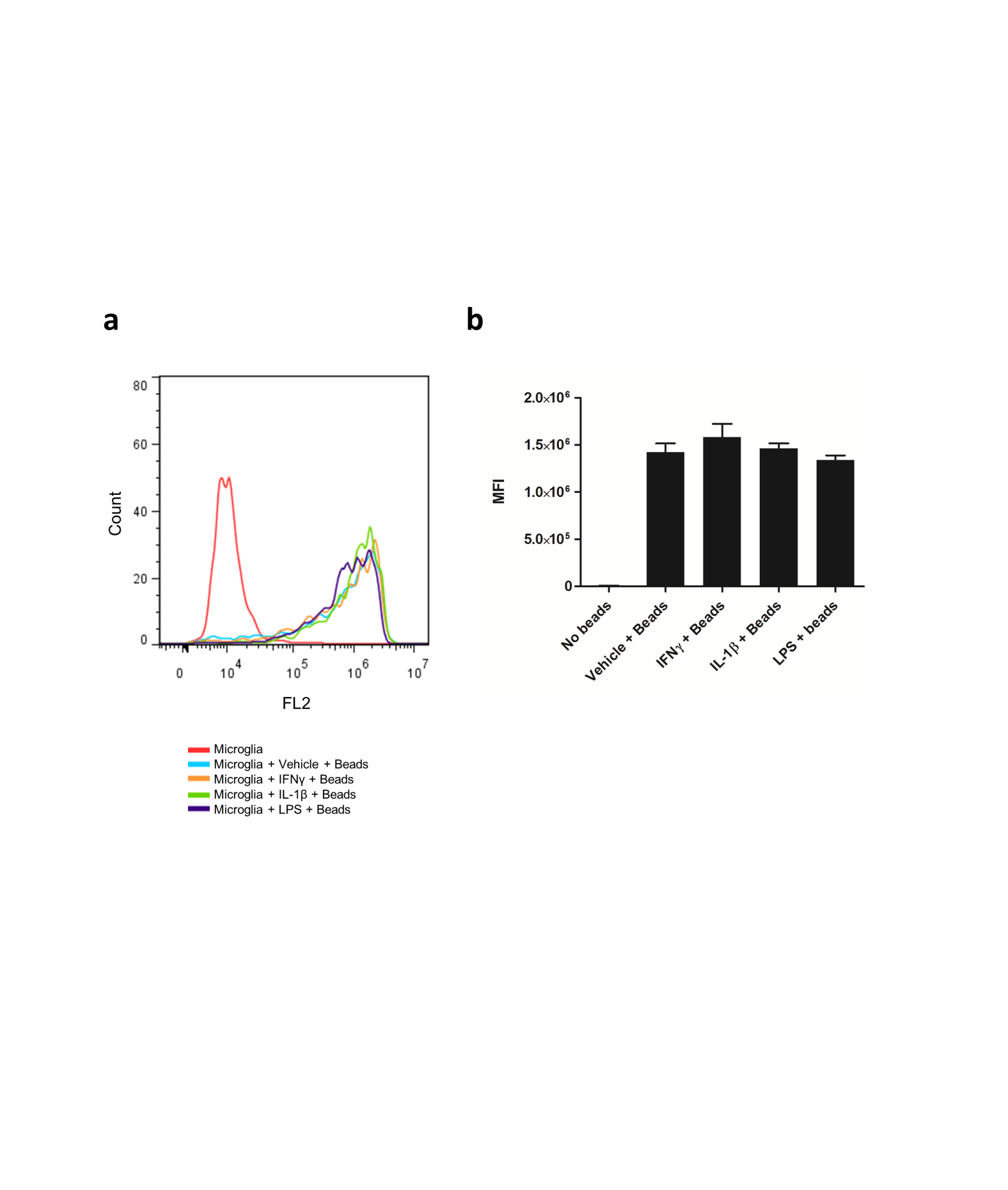
**

**Figure S4. Immune stimuli do not affect microglial phagocytosis.** Microglia were treated with vehicle (0.1% BSA in PBS) or 10 ng/mL IL-1β, IFNγ or LPS for 24 hours. For the final two hours of treatment cells were incubated with fluorescent latex beads (1 µm diameter) and phagocytosis determined via flow cytometry. One representative plot from three independent cases (one epilepsy and two GBM) is shown (a) and the mean fluorescence intensity of FL2 channel determined from n = 3 cases (b).

**
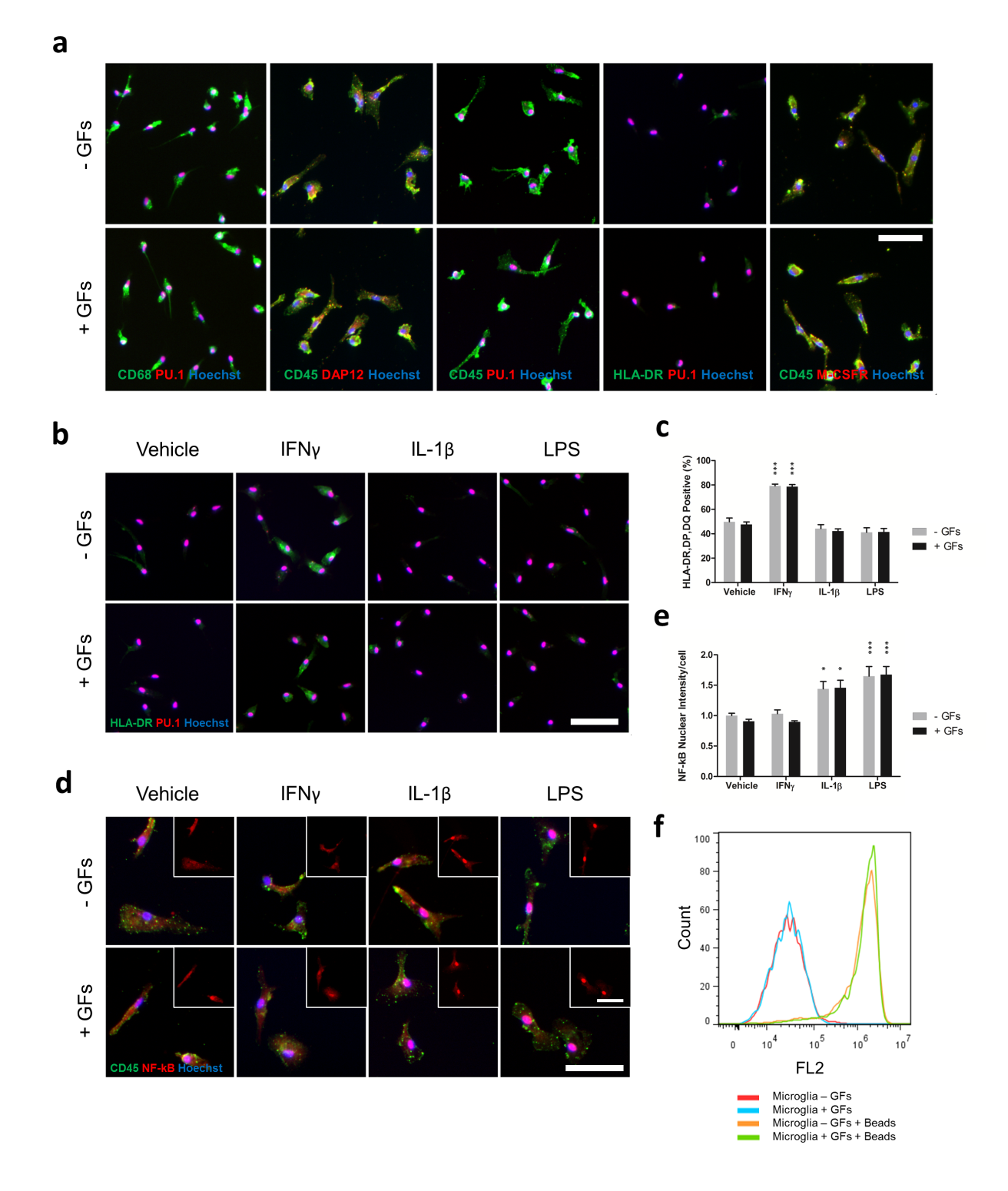
**

**Figure S5. NPC growth factors are not required for microglial survival but do not perturb microglial phenotype.** Microglia were cultured from a sample of cerebellum obtained during tumour resection (a, b, c, d, e) or a tumour in superior frontal cortex (f) as per the microglial isolation protocol with slight modifications. At the final stage of processing, samples were split in half and cultured in the presence or absence of 40 ng/mL FGF-2, 40 ng/mL EGF and 2 µg/mL heparin. Microglial phenotype in each culture was examined using microglial markers PU.1, CD45, HLA-DR, CD68, DAP12 and M-CSFR (a). Microglial activation was determined by HLA-DR immunostaining after 24 hours (b, c) or nuclear NF-kB immunostaining after one hour (d, e) with 10 ng/mL IL-1β, IFNγ, LPS or vehicle (0.1% BSA in PBS). Microglial phagocytosis in each condition was determined by flow cytometry after a two hour incubation with fluorescent beads (1 µm diameter; f). Scale bar = 50 µm. * = p < 0.05, *** p < 0.001.
